# Effect of COPD on Inflammation, Lymphoid Functions and Progression-Free Survival during First-Line Chemotherapy in Advanced Non-small Cell Lung Cancer

**DOI:** 10.1007/s12253-019-00661-w

**Published:** 2019-05-14

**Authors:** Márton Szentkereszty, Zsolt István Komlósi, Gergő Szűcs, Gábor Barna, Lilla Tamási, György Losonczy, Gabriella Gálffy

**Affiliations:** 1 Department of Pulmonology, Semmelweis University, Diós árok 1/C, Budapest, H-1121 Hungary; 2 1st Department of Pathology and Experimental Cancer Research, Semmelweis University, Üllői út 26, Budapest, H-1085 Hungary

**Keywords:** Advanced NSCLC, COPD, Inflammation, Lymphopenia, VEGF, MDSC, Neutrophils, CRP, T cell exhaustion

## Abstract

Chronic obstructive pulmonary disease (COPD) is a common comorbidity of non-small cell lung cancer (NSCLC). COPD is characterized by systemic inflammation and lymphocyte dysfunction, mechanisms that are also known to accelerate progression of advanced (IIIB-IV) stage NSCLC. We aimed to find out whether COPD exerts an influence on tumor induced inflammatory and lymphoid responses and progression-free survival (PFS) after first-line treatment in advanced NSCLC. Patients suffering from NSCLC (*n* = 95), COPD (*n* = 54), NSCLC+COPD (*n* = 80) and healthy controls (*n* = 60) were included. PFS, neutrophil granulocyte and lymphocyte cell counts were recorded. Serum IFNγ, TNFα, VEGF concentrations were measured by using multiplex cytometric bead-based immunoassay. Prevalence of myeloid-derived suppressor cell populations (MDSC-s), and signs of T cell exhaustion were tested by using flow cytometry. Median PFS increased in the NSCLC+COPD group compared to NSCLC patients without COPD (7.4 vs 4.9 months, *p* < 0.01). NSCLC+COPD patients had 1.7 times (1.2–2.4) more likely to have longer PFS compared to NSCLC patients without COPD (Cox analysis, *p* < 0.01). Neutrophil cell counts, CRP, IFNγ and TNFα concentrations were all reduced in NSCLC+COPD (all *p* < 0.05 vs NSCLC). NSCLC+COPD was also associated with reduced serum IL-10 concentration and increased granzyme-B positive CD8 cell counts compared to NSCLC without COPD. The effects of VEGF and MDSC-s on systemic inflammation appeared to be blunted by COPD in patients suffering from advanced NSCLC. Concomitant COPD moderates tumor-induced inflammation and supports some effector lymphoid functions and thereby may be an independent positive predictive factor of longer PFS after first-line therapy in advanced NSCLC.

## Background

Progression-free survival (PFS) is the length of time during and after the treatment of a disease, such as cancer, that a patient lives without worsening the disease. In a clinical trial, measuring the PFS is one way to see how well a new treatment works [1]. In advanced non-small cell lung cancer (NSCLC) standard first-line treatment is induction and maintanance therapy with cytotoxic and/or biologically targeted agents. Prognosis depends on both tumor- and host-related factors. Stages of primary tumor, lymph node and remote metastasis are major tumor-related, while weight loss and performance state are major host-related factors [2]. Performance state, pain, weight loss and functional disability are considered manifestations of tumor-induced inflammation [3], moreover, lower than median neutrophil cell counts has been demonstrated to be a strong positive predictive biomarker of longer PFS during first-line treatment in advanced NSCLC [4]. On the other hand, T cell exhaustion [5] and lower total lymphocyte cell counts [6] are signs of negative prognosis in advanced cancer. The neutrophil/lymphocyte ratio has been repeatedly shown to be a strong indicator of PFS and overall survival (OS) in advanced NSCLC [7, 8]. Although advanced NSCLC is the cancer of heavy smokers and the elderly most of whom suffer from comorbidities [9], there has been a lack of information how comorbidities influence PFS during and after first-line treatment. The influence of a very frequent comorbidity, chronic obstructive pulmonary disease (COPD) has been studied in only a few previous series [10–14]. PFS [10] and OS [11, 12] remained unchanged by COPD after first-line treatment, while in two other studies PFS was observed to become prolonged by concomitant COPD in advanced NSCLC [13, 14]. Mark et al. [14] reported that in advanced NSCLC, the presence of COPD was associated with enhanced efficacy of immune checkpoint inhibitors as PFS was longer (*p* = 0.0168) in NSCLC with COPD versus no COPD (*n* = 125). The issue remains clinically relevant since approximately 50% of NSCLC patients suffer from COPD [9, 15], which itself is a state of chronic inflammation and lymphoid dysfunction [16].

In advanced NSCLC circulating vascular endothelial growth factor (VEGF) concentration increases [17–19]. In addition to angiogenesis VEGF was shown to play a role in modulation of myelopoiesis including the formation of myeloid-derived suppressor cells (MDSC-s) which have been known to contribute to tumor-induced inflammation and immunosuppression [20, 21]. One manifestation of immunosuppression in cancer is appearance of exhausted T cells (CD4^+^T^exh^and CD8^+^T^exh^) within tumor tissue [22] and in the systemic circulation [23, 24]. VEGF, MDSC-s, inflammation and the appearance of T^exh^ cells have all been reported to be present also in COPD [25, 26], not only cancer.

Therefore, in the present study, we compared PFS, myeloid and lymphoid responses, circulating VEGF, MDSC-s, as well as effector and exhausted T cell counts in patients suffering from advanced NSCLC with or without COPD.

## Methods

### Patients

All patients were taken care at the Department of Pulmonology, Semmelweis University Medical Center between November 2015 and March 2017. Written informed consent was obtained from every patient and permission was obtained from the Institutional Ethics Committee of Semmelweis University Clinical Center (#238–2/2015). The total number of pathologically confirmed, stage IIIB-IV (advanced) NSCLC patients was 95, while the number of NSCLC patients with concomitant COPD (NSCLC+COPD) was 80. Sixty healthy smokers and 54 COPD patients were included as controls. In addition to general clinical parameters plasma IFNγ, TNFα and interleukin-(IL-)10, or serum VEGF and circulating monocytic (M-) and granulocytic (G-) myeloid-derived suppressor cells (MDSC-s), or subpopulations of CD4 and CD8 T cells positive for IFNγ, granzyme B, PD1 or CTLA4 were quantitated. Since concomitant COPD was observed to markedly alter the relationship between VEGF and MDSC-s, as well as neutrophil and lymphocyte cell counts, more bevacizumab+paclitaxel+carboplatin treated adenocarcinoma patients (without or with COPD) were included. Stage of NSCLC according to Quint [27] and performance state according to Eastern Cooperative Oncology Group (ECOG) [28] were determined. Treatment effects on primary tumor, metastatic lymph node(−s) or remote metastasis were evaluated according to RECIST 1.1. Progression-free survival (PFS) was calculated as the time from start of first-line treatment to disease progression or death. Progression was controlled every 3 week by clinical examination and chest X-ray and if any signs or symptoms have raised suspision of progression, CT, PET-CT and/or MRI were performed. Chest and upper abdominal CT was performed every 3 months minimum. Overall survival (OS) was estimated from start of oncologic treatment until exitus. COPD was diagnosed according to the criteria of the Global Initiative for Chronic Obstructive Lung Disease 2017 [29]. COPD patients were in GOLD stage II-III. Lung function results and the bronchoscopic state of large airways of each NSCLC patient were cautiously analysed to exclude those, who had airway obstruction of cancerous or other, not COPD related cause.

None of the included patients and subjects used systemic steroid or antibiotics at least two months before the investigation. All COPD patients (with or without NSCLC) were treated by maintanance inhalational long-acting muscarinerg antagonist (LAMA), or long-acting β2 agonist (LABA), LAMA+LABA, or inhalational corticosteroid+LABA. Subjects were excluded if they had a history of another malignancy or other diseases associated with systemic inflammation or immunodeficiency. None of the COPD patients (with or without cancer) suffered from acute exacerbation.

Peripheral venous blood was collected at the time of diagnosis, before the administration of any cytostatic or targeted anticancer drug. Body mass index (BMI) and smoking history was calculated and the plasma concentration of absolute neutrophil cell counts, monocyte cell counts, lymphocyte cell counts, platelets and concentration of hemoglobin (Hb) and C-reactive protein (CRP) were measured. Post-bronchodilator forced expiratory volume in 1 s (FEV_1_) and forced vital capacity (FVC) were tested by Total Body Plethysmograph (Piston, Budapest, Hungary).

### Flow Cytometry

Peripheral venous blood samples were collected in sodium heparin tubes (Vacutainer, Becton Dickinson, Franklin Lakes, USA). Cell preparations and flow cytometric analyses were performed according to the protocols described earlier [30]. Briefly, peripheral blood mononuclear cells (PBMCs) were isolated by Ficoll (Biochrom, Berlin, Germany) density gradient centrifugation within 3 h of sample collection. MDSC measurements were performed right after the PBMC isolation. PBMC samples were frozen and stored in biobank until the exhausted T cell measurements. For cryopreservation, PBMCs were resuspended in freezing medium (10% DMSO and 45% fetal bovine serum in complete RPMI 1640) and stored −80 °C until stimulation and flow cytometric measurements. For the CTLA-4 and intracellular IFN-γ measurements defrosted cells were cultured in RPMI 1640 medium supplemented with 1 mM sodium pyruvate (Sigma), 1% MEM nonessential amino acids and vitamins (Sigma), 2 mM L-glutamine (Sigma), 100 U/ml penicillin, 100 μg/ml streptomycin (Sigma), 100 μg/ml kanamycin (Gibco) and 10% heat-inactivated FCS (Sigma) in 24 well plates, and stimulated with PMA (25 ng/ml; Sigma) and ionomycin (1 μg/mL; Sigma) for 6 h in the presence of brefeldin A (10 μg/ml; Sigma) for the final 2 h. Cytofix/Cytoperm Fixation and Permeabilization Kit (Beckton Dickinson) were used for cell permeabilization and intracellular staining procedure. Flow cytometry data were acquired on FACSAria (Beckton Dickinson) and Navios (Beckman Coulter, Brea, USA) instruments, and were analyzed with Kaluza software (Beckman Coulter).

The following anti-human antibodies were used for flow cytometry measurements: fluorescein isothiocyanate (FITC)-conjugated anti-CD15 (HI98), phycoerythrin (PE)-conjugated anti-CD33 (WM53) and PE-conjugated anti-IFN-γ (4S.B3), PE/Dazzle 594-conjugated anti-HLA-DR (L234), peridinin chlorophyll protein-cyanine 5.5 (PerCP-Cy5.5)-conjugated anti-CD66b (G10F5), PE-indotricarbocyanine (Cy7)-conjugated anti-CD14 (M5E2), allophycocyanin (APC)-conjugated anti-CD11b (ICRF44) and isotype-matched control antibodies conjugated to PE, PE/Dazzle 594 and Alexa Fluor 647 (MOPC-21; all from BioLegend, San Diego,USA); FITC-conjugated anti-CD4 (sk3), PE/Dazzle 594-conjugated anti-CD8 (sk1), PerCP-Cy5.5-conjugated anti-CD3 (UCHT1), PE-Cy7-conjugated anti-CTLA-4 (CD152; L3D10), Alexa Fluor 647-conjugated anti-Granzyme B (GB11), APC-Cy7-conjugated anti-PD-1 (CD279, EH12.2H7; all from Sony Biotechnology, San Jose, USA); isotype-matched control antibodies conjugated to FITC, PerCP-Cy5.5, PE-Cy7, APC and APC-Cy7 (MOPC-21; all from Beckton Dickinson); APC-eFluor 780 anti-human CD19 (HIB19), CD3 (UCHT1), CD56 (CMSSB) and isotype-matched control antibodies conjugated to APC-eFluor 780 (P3.6.2.8.1; all from eBioscience, Affymetrix, Santa Clara, USA). A fixable viability dye (eFluor 780, termed as e-780 on figure; eBioscience) was used for dead cell discrimination.

In order to assess the level of exhaustion in T cell populations the spontaneous cell surface expression of PD-1, and the IFN-γ production as well as the CTLA-4 expression after PMA/ionomycin stimulation both in CD3 + CD4+ T helper and CD3 + CD8+ T killer cells were measured. The intracellular granzyme B content was also measured in CD3 + CD8+ T killer cells [22].

**Fig. 1:**
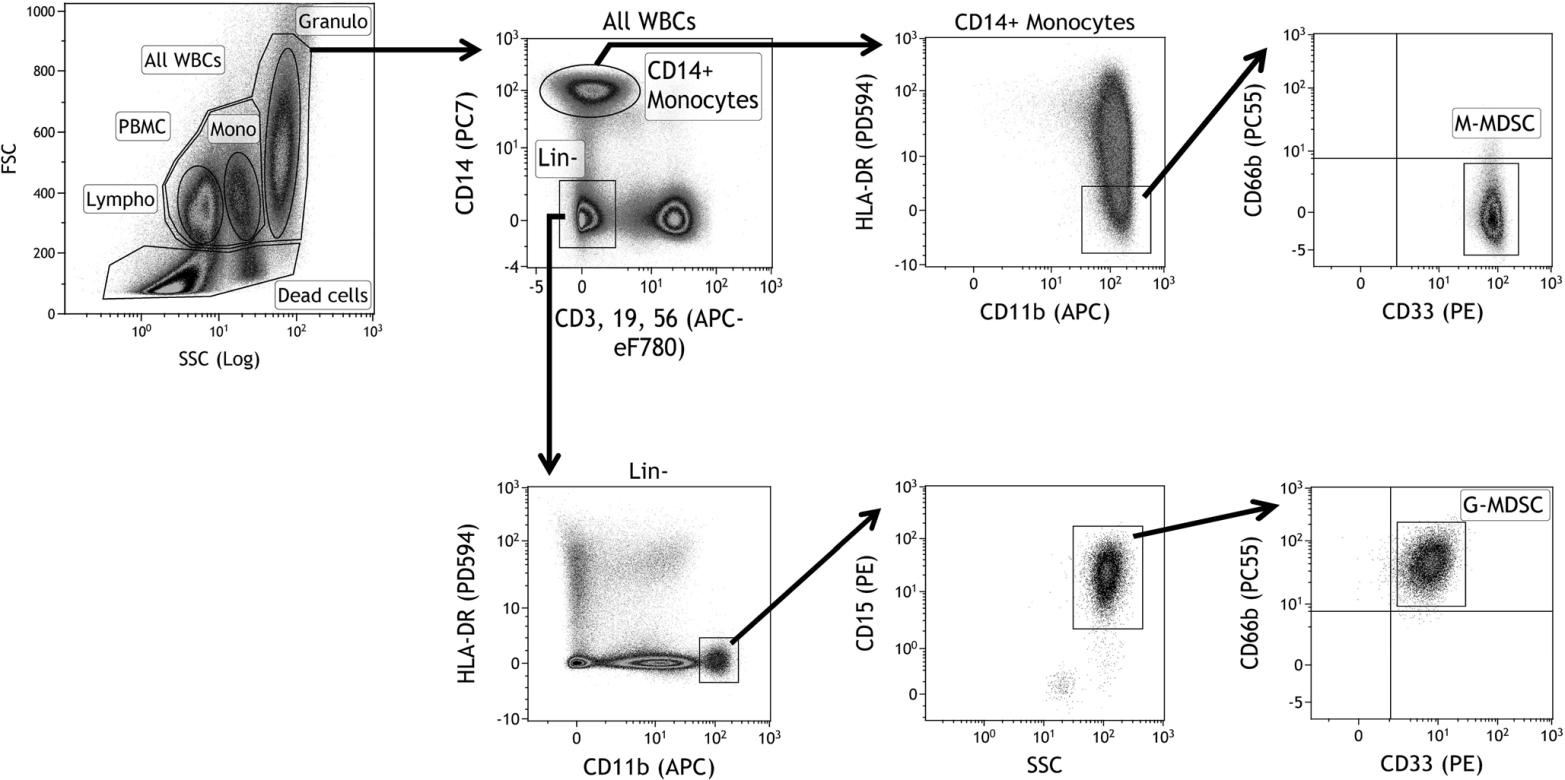
Flow cytometric identification of myeloid-derived suppressor cells

MDSC populations were defined according to Bronte et al. [31] as monocytoid-(M-)MDSC: CD3^−^CD19^−^CD56^−^CD14^+^HLA-DR^−^CD11b^+^CD33^high^ and granulocytoid-(G-)MDSC:Lin^−^HLA-DR^−^CD11b^high^CD15^high^CD33^low^CD66b^+^ cells (Fig. 1). Lineage markers (Lin) were: CD14, CD3, CD19, CD56. Peripheral blood samples after density gradient centrifugation, especially in cancer patients, were containing low-density granulocytes besides mononuclear cells. Therefore the bona fide mononuclear PBMCs (lymphocytes and monocytes) identified by size and complexity (on FSC-SSC plot) were gated, and the M-MDSCs were expressed as percent of the gated PBMCs. G-MDSCs were expressed as percent of all WBCs in samples after density gradient centrifugation (containing PBMCs and low-density granulocytes).

### Analysis of Cytokines

Concentrations of IFN, TNF, IL-10 were measured in serum by multiplex cytometric bead-based immunoassays (Bio-Plex system, Bio-Rad, Hercules, CA, USA). Concentration of VEGF in serum samples were measured by commercially available ELISA kit (R&D Systems, Minnesota, USA). The assays were performed according to instructions of manufacturers.

### Statistics

Categorical data were compared by Pearson χ^2^ test (or Fisher exact test as needed). Continuous data were examined by normality test. Continuous variables with normal distribution were presented as mean ± standard error of the mean (SEM) or as median with {interquartile range} where normal distribution was absent. Continuous data were compared with one-way ANOVA and Pearson test if distribution was normal or with Kruskal-Wallis test and Spearman test if the sample distribution was asymmetrical. We used Tukey post-hoc test for one-way ANOVA and Dunn’s test for Kruskal-Wallis. All the *p* values are two-sided, and *p* < 0.05 was considered statistically significant. Differences between groups considering PFS and OS were estimated using Kaplan-Meier method, and log-rank test was used for comparison of outcomes. Independent prognostic variables of PFS were assessed using Cox proportional hazards regression analysis. IBM SPSS Statistics 22 (SPSS Inc., Chicago, IL, USA) and GraphPad Prism 5.01 (GraphPad Software Inc., San Diego, CA, USA) were used for statistical analysis.

## Results

### Clinical Data

Age, gender and smoking history were similar in patient groups (COPD, NSCLC and NSCLC+COPD) (Table 1). Among the 95 NSCLC patients 49 were ex- and 46 were current smokers. Among the 80 NSCLC+COPD patients 43 were ex- and 37 were current smokers (*p* > 0.05 vs NSCLC). All women patients were in postmenopausal age. Most NSCLC patients (without or with COPD) were in oncological stage IV. In both NSCLC groups there were more non-squamous than squamous histologic types, but the ratio of the various histologies was similar within the two groups. The FEV_1_/FVC ratio was less than 70% and FEV_1_ was reduced in the two COPD groups, while BMI was smaller in the two NSCLC groups. Since relatively more non-squamous NSCLC patients were included, more patients received bevacizumab-containing chemotherapy. The ratio of the various therapeutic modalities including radiotherapy were similar in the NSCLC and the NSCLC+COPD groups.

**Table 1:** Clinical data, progression-free and overall survival

Group	Control *n* = 60	COPD *n* = 54	p vs Control	NSCLC *n* = 95	p vs Control	NSCLC+COPD n = 80	p vs		
							Control	COPD	NSCLC
male^1^	15	27		54		50			
female	45	27		41		30			
age (years)	54.3 ± 0.9	63.2 ± 1.0	<.001	63.3 ± 0.9	<.001	65.3 ± 0.9	<.001	ns	ns
smoking (pack-year)	28 {15–45}	42 {31–50}	<.001	40 {28–48}	ns	40 {30–50}	<.05	ns	ns
oncologic stage^1^									
IIIB	–	–		12		14			
IV	–	–		83		66			
adenocarcinoma^1^	–	–		73		60			
squamous cell cc.	–	–		22		20			
ECOG state^1^									
0	–	–		56		45			
1	–	–		35		32			
2	–	–		4		3			
FEV_1_ (% of pred.)	103.1 ± 2.4	46.1 ± 2.4	<.001	78.8 ± 2.3	<.001	62.0 ± 2.3	<.001	<.001	<.001
FEV_1_/FVC	81.7 ± 1.0	51.1 ± 1.9	<.001	79.2 ± 1.6	ns	62.5 ± 2.1	<.001	<.001	<.001
BMI (kg/m^2^)	26.9 ± 1.2	28.3 ± 1.1	ns	26.2 ± 0.8	ns	24.2 ± 0.8	ns	<.05	ns
treatment^1^									
platinum+gemcitabine	–	–		14		11			
platinum+pemetrexed	–	–		15		13			
platinum+taxan	–	–		14		9			
platinum+taxan+bevac.	–	–		48		40			
other ^¤1^	–	–		4		7			
radiotherapy	–	–		32		20			
no radiotherapy	–	–		63		60			
WBC (G/L)	7.2 ± 0.2	9.0 ± 0.3	<.05	11.9 ± 0.5	<.001	10.3 ± 0.4	<.001	ns	<.05
neu count (G/L)	4.3 ± 0.2	5.7 ± 0.3	ns	9.2 ± 0.4	<.001	7.5 ± 0.3	<.001	<.01	<.01
ly count (G/L)	2.3 ± 0.1	2.4 ± 0.1	ns	1.7 ± 0.1	<.001	1.9 ± 0.1	ns	<.05	ns
neu/ly ratio	1.96 ± 0.08	2.72 ± 0.23	ns	7.41 ± 0.76	<.001	4.54 ± 0.27	ns	<.001	< .01
monocytes (G/L)	0.42 ± 0.02	0.56 ± 0.03	ns	0.62 ± 0.03	<.001	0.67 ± 0.04	<.001	ns	ns
hemoglobin (g/L)	143 ± 2	148 ± 2	ns	133 ± 2	<.001	137 ± 2	ns	<.01	ns
platelets (G/L)	240 ± 7	257 ± 10	ns	346 ± 17	<.001	324 ± 12	<.001	<.01	ns
CRP (mg/L)	3 {2–5}	4 {3–8}	ns	17 {6–59}	<.001	8 {4–19}	ns	ns	<.001
median PFS (month)	–	–		4.9		7.4			<.01
median OS (month)	–	–		11.0		16.9			ns

BMI: body mass index, WBC: white blood cell count, neu: neutrophil, ly: lymphocyte, CRP: C-reactive protein concentration; ^1^no statistical difference among various groups, ^¤^other treatments: gemcitabine (*n* = 4), docetaxel (n = 3), platinum+vinorelbin (*n* = 2), platinum+etoposid (n = 2)

Frequency of comorbidities within NSCLC (*n* = 95) and NSCLC+COPD (*n* = 80) patients were similar: hypertension (51/95 and 47/80), ischaemic heart disease (12/95 and 13/80), diabetes mellitus (14/95 and 17/80) and cachexia (17/95 and 16/80), respectively (all *p* > 0.05). None of cancer patients had creatinine clearance less than 60 mL/min.

### Routine Laboratory Data

With the exception of a moderately increased white blood cell count (WBC) laboratory data remained unchanged in COPD versus control patients (Table 1). In NSCLC the absolute neutrophil cell count was increased, but less so in case of concomitant COPD. The lymphocyte cell count was reduced in NSCLC vs healthy controls and in NSCLC+COPD vs COPD patients. The neutrophil/lymphocyte ratio was increased in NSCLC and relatively reduced in NSCLC+COPD. CRP concentration was also increased in NSCLC but reduced by concomitant COPD. Based on neutrophil, neutrophil/lymphocyte ratio and CRP data inflammation induced by advanced NSCLC became reduced by concomitant COPD (Table 1).

### Cytokines

The concentration of proinflammatory cytokines IFNγ and TNFα were found to be strongly increased in NSCLC but reduced in those patients who had concomitant COPD. IL-10, the cytokine fostering immunologic tolerance of cancer was high in NSCLC, but lower (in the control range) in NSCLC+COPD (Table 2).

**Table 2:** Cytokines, vascular endothelial growth factor, myeloid-derived suppressor cells and T cell subpopulations

Group	Controls	COPD	p vs Control	NSCLC	p vs Control	NSCLC+COPD	p vs		
							Control	COPD	NSCLC
cytokines	*n* = 24	*n* = 26		*n* = 29		*n* = 19			
IFNγ (pg/mL)	10.8 ± 1.9	36.6 ± 5.9	ns	83.0 ± 19.9	<.001	32.7 ± 8.1	ns	ns	<.05
TNFα (pg/mL)	7.6 ± 1.2	21.8 ± 3.7	ns	38.5 ± 6.3	<.001	19.0 ± 5.3	ns	ns	<.05
IL-10 (pg/mL)	0.85 ± 0.08	1.49 ± 0.17	ns	2.97 ± 0.45	<.001	1.45 ± 0.22	ns	ns	<.01
VEGF, MDSC	*n* = 27	*n* = 17	*n* = 21	n = 19					
plasma VEGF (pg/mL)	545 ± 43	526 ± 67	ns	1123 ± 159	<.01	1243 ± 158	<.001	<.001	ns
M-MDSC/CD14^+^ (%)	3.77 ± 0.55	3.71 ± 0.77	ns	9.71 ± 1.44	<.001	7.88 ± 0.84	<.01	<.05	ns
G-MDSC/all WBCs (%)	1.35 ± 0.24	2.55 ± 0.58	ns	4.48 ± 0.81	<.001	2.27 ± 0.45	ns	ns	<.05
T cell subpopulations	n = 9	*n* = 11		*n* = 14		*n* = 13			
CD3^+^CD4^+^(x10^8^cell/mL)	12.49 ± 0.99	10.15 ± 1.61	ns	7.43 ± 1.61	ns	9.73 ± 1.89	ns	ns	ns
IFNγ^+^ (x10^7^cell/mL)	13.01 ± 2.68	11.53 ± 2.25	ns	9.45 ± 1.98	ns	16.95 ± 4.32	ns	ns	ns
granz-B^+^ (x10^7^cell/mL)	3.89 ± 0.63	9.79 ± 2.52	ns	6.95 ± 2.84	ns	8.70 ± 5.31	ns	ns	ns
PD1^+^ (x10^8^cell/mL)	1.38 ± 0.20	1.29 ± 0.24	ns	1.07 ± 2.53	ns	1.51 ± 0.45	ns	ns	ns
CTLA4^+^ (x10^7^cell/mL)	4.56 ± 0.80	4.21 ± 1.11	ns	3.18 ± 0.64	ns	4.96 ± 1.42	ns	ns	ns
CD3^+^CD8^+^(x10^8^cell/mL)	4.11 ± 0.49	5.34 ± 1.05	ns	3.10 ± 0.66	ns	4.77 ± 0.93	ns	ns	ns
IFNγ^+^ (x10^8^cell/mL)	1.36 ± 0.34	1.52 ± 0.42	ns	1.24 ± 0.29	ns	2.06 ± 0.50	ns	ns	ns
granz-B^+^(x10^8^cell/mL)	1.94 ± 0.32	2.61 ± 0.64	ns	1.48 ± 0.40	ns	4.35 ± 1.12	ns	ns	<.05
PD1^+^ (x10^7^cell/mL)	8.35 ± 1.07	9.16 ± 2.26	ns	7.15 ± 1.51	ns	12.65 ± 3.37	ns	ns	ns
CTLA4^+^ (x10^6^cell/mL)	7.19 ± 1.40	5.91 ± 1.30	ns	5.59 ± 1.38	ns	9.01 ± 2.08	ns	ns	ns

VEGF: vascular endothelial growth factor, M-MDSC: monocytic myeloid-derived suppressor cells, G-MDSC: granulocytic myeloid-derived suppressor cells, granz-B: granzyme B;

### VEGF and MDSC-S

**Fig. 2:**
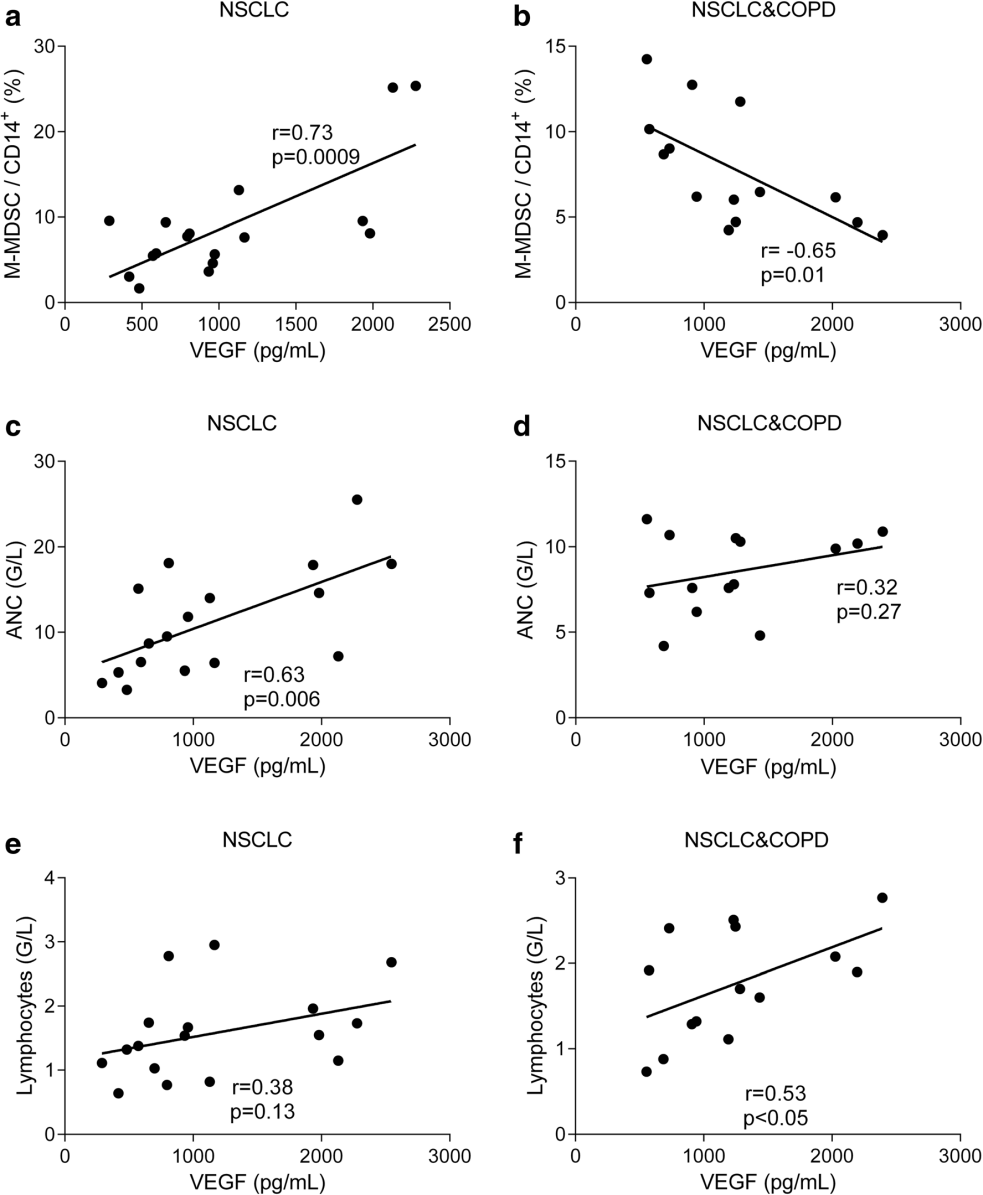
Relationship between serum VEGF and M-MDSC (**a**, **b**), neutrophil granulocyte (**c**, **d**) and lymphocyte cell counts (**e**, **f**) in NSCLC (**a**, **c**, **e**) and NSCLC+COPD (**b**, **d**, **f**) patients

Since earlier studies indicated that in NSCLC the synthesis of VEGF was increased and VEGF fostered the formation of MDSC-s [17–19, 32, 33], in the next subgroups VEGF and MDSC-s were measured. Serum VEGF as well as peripheral blood M- and G-MDSC-s were increased in NSCLC (Table 2). When NSCLC was combined with COPD, the M-MDSC fraction remained increased, but the G-MDSC fraction normalized. The relationships between serum concentration of VEGF and sizes of various cell populations were analysed. Higher serum VEGF had a direct relationship with M-MDSC-s in NSCLC (r = 0.73, *p* < 0.001, Fig. 2a), but not in NSCLC+COPD patients, in whom this relationship became inverse (r = −0.65, *p* = 0.01, Fig. 2b). Thus, concomitant COPD reduced G-MDSC-s and inverted the effect of VEGF on M-MDSC-s, which have been known to exert both proinflammatory and immunosuppressive effects in NSCLC [21, 33]. There was no relationship between serum VEGF and G-MDSC-s in either groups.

### VEGF and Blood Cells

Higher VEGF was, however, also associated with higher neutrophil cell count in NSCLC (r = 0.63, *p* = 0.006, Fig. 2c), but not in NSCLC+COPD (r = 0.32, *p* = 0.27, Fig. 2d). On the other hand, there was no relationship between VEGF and lymphocyte cell count in NSCLC (r = 0.38, *p* = 0.13, Fig. 2e), while higher VEGF was accompanied with higher lymphocyte cell count in NSCLC+COPD patients (r = 0.53, *p* < 0.05, Fig. 2f). Therefore, VEGF-associated mechanisms of both neutrophilia and lymphocytopenia were reduced by concomitant COPD.

### Exhausted and Effector CD4 and CD8 Cells

In order to evaluate the functional state of circulating T cells, in further subgroups the CD4 and CD8 fractions with intracellular IFNγ and granzyme-B content, as well as those with surface expression of PD1 and CTLA4 immune checkpoint molecules were measured (Table 2). These lymphocyte subpopulations were of similar size within both CD4 and CD8 cells, which indicated the absence of systematic T cell exhaustion in either cancer groups. Moreover, the abundance of granzyme-B positive CD8 cells was augmented (more than 90% of all CD8 cells) in NSCLC+COPD patients as compared to those suffering from only NSCLC (about 50% of CD8 cells). Thus, NSCLC+COPD patients had reduced IL-10 concentration and circulating G-MDSC-s (both normal, see Table 2), as well as more effector CD8 cells. Moreover, only NSCLC+COPD patients presented a direct relationship between VEGF and lymphocyte count, but an indirect relationship between VEGF and M-MDSC-s.

**Table 3:** Cox-regression analysis of factors influencing progression-free survival (*n* = 175)

Variable	n (%)	Progression-free survival			
		Univariate analysis		Multivariate analysis*	
		Median (mo)	p value	HR (95% CI)	p value
age					
<65 years	93 (53)	5.7	ns	0.9 (0.6–1.2)	0.542
≥65 years	82 (47)	5.3	–	–	–
gender					
Male	104 (59)	5.3	ns	1.1 (0.8–1.6)	0.497
Female	71 (41)	5.8	–	–	–
COPD					
No	95 (54)	4.9	0.0017	1.7 (1.2–2.4)	0.002
Yes	80 (46)	7.4	–	–	–
ECOG					
0	101 (58)	6.1	–	–	–
1–2	74 (42)	4.4	ns	1.3 (0.9–1.8)	0.123
histology					
Adenocarcinoma	133 (76)	5.8	ns	0.7 (0.5–1.1)	0.180
Squamous cell carcinoma	42 (24)	5.7	–	–	–
stage					
III/B	26 (15)	11.5	0.0422	1.6 (0.9–2.7)	0.093
IV	149 (85)	5.3	–	–	–
radiotherapy					
Yes	52 (30)	5.3	–	–	–
No	123 (70)	5.9	ns	0.9 (0.7–1.4)	0.774

*Cox-regression model also included the type of chemotherapy, which did not influence PFS (not shown). HR = hazard ratio, CI = confidence interval, NS = not significant

### Progression-Free and Overall Survival

**Fig. 3:**
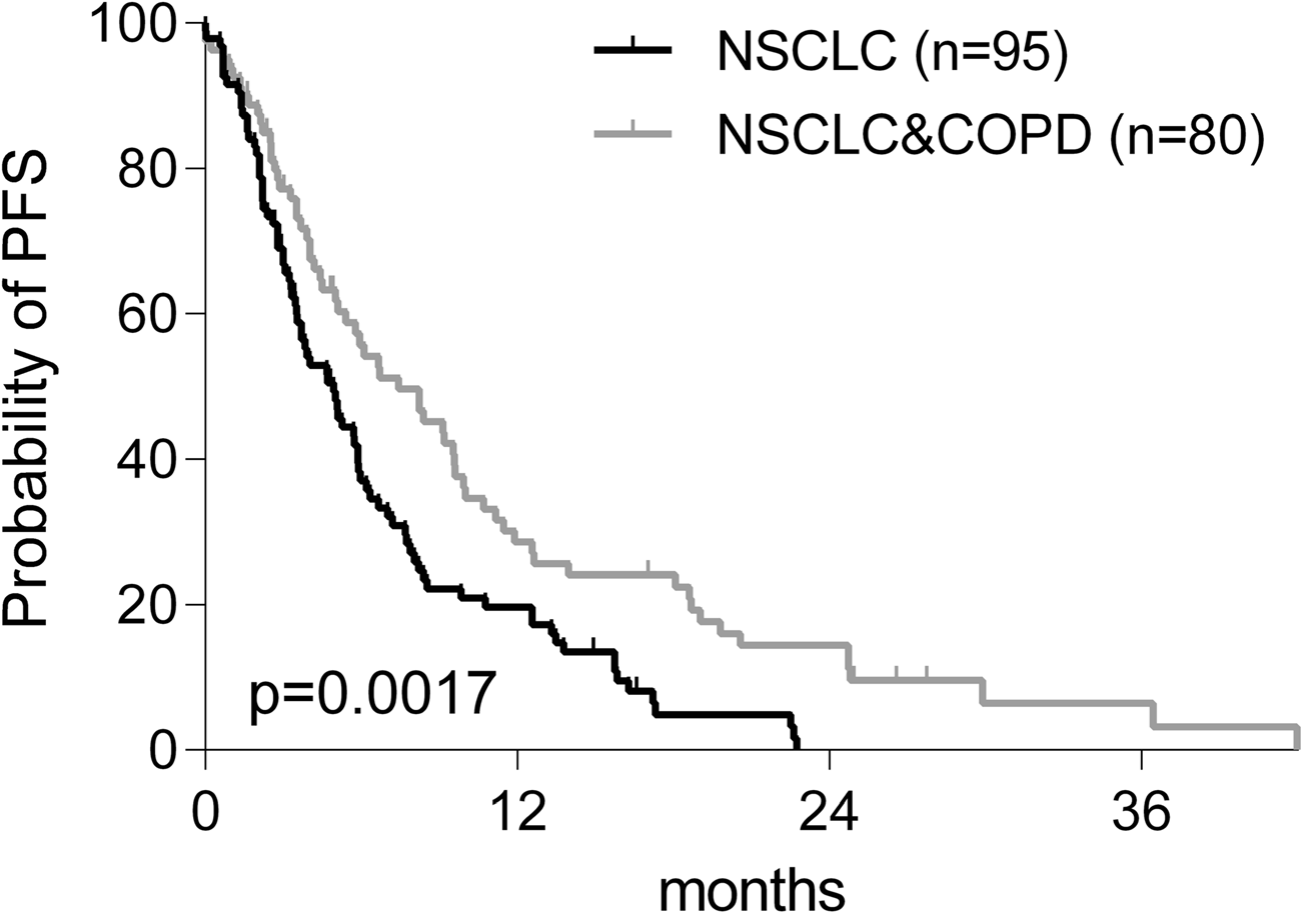
Kaplan-Meier analysis of progression-free survival in NSCLC and NSCLC+COPD patients

Tumor-induced inflammation is a strong, negative prognostic factor of survival in advanced cancers including NSCLC [3, 4]. In line with these observations of other authors, in the present study PFS was prolonged in NSCLC+COPD patients vs those suffering from NSCLC without COPD. Median PFS was 7.4 months in NSCLC+COPD vs 4.9 months in NSCLC (*p* < 0.01). OS was 16.9 and 11.0 months in NSCLC+COPD and NSCLC, respectively (*p* > 0.05, Table 1). Figure 3 shows the PFS increasing effect of concomitant COPD as confirmed by Kaplan-Meier analysis (*p* = 0.0017). By Cox proportional hazards regression analysis the hazard ratio of longer PFS in NSCLC+COPD vs only NSCLC was 1.70, (CI: 1.2–2.4, *p* = 0.002, Table 3). Thus, concomitant COPD was found to be an independent positive predictive factor of longer PFS during first-line treatment of advanced NSCLC.

### Effects of Concomitant COPD in Bevacizumab+Chemotherapy Treated Advanced NSCLC Patients

According to earlier findings, lower pretreatment VEGF in plasma [17, 34] or serum [18, 19] predicted longer survival in advanced NSCLC. Based on these and the present finding that concomitant COPD altered the relationships between VEGF and MDSC-s, neutrophils and lymphocytes, it seemed relevant to find out whether the PFS prolonging effect of concomitant COPD was altered when compared between homogenously bevacizumab+chemotherapy treated patients. Therefore, two further, otherwise matching, non-squamous, stage IIIB-IV NSCLC groups (one without and one with COPD) were recruited. All patients have received bevacizumab combined with carboplatin+paclitaxel. Patients with concomitant COPD (*n* = 35) had again lower neutrophil, higher lymphocyte cell count and lower neutrophil/lymphocyte ratio (all *p* < 0.05), as well as longer PFS (*p* = 0.049) than those NSCLC patients who had no COPD (*n* = 39). Therefore, homogenous anti-VEGF treatment did not change the antiinflammatory and PFS prolonging effects of concomitant COPD (Table 4).

**Table 4:** Clinical data of stage IIIB-IV, non-squamous NSCLC patients received bevacizumab with carboplatin+paclitaxel

variable	NSCLC	NSCLC+COPD	p vs
	n = 39	n = 35	NSCLC
Male^1^	20	17	
Female	19	18	
Age (years)	62 ± 2	65 ± 1	ns
Oncologic stage^1^			
IIIB	4	4	
IV	35	31	
ECOG state^1^			
0	27	23	
1	11	11	
2	1	1	
FEV_1_ (% of pred.)	81 ± 3	62 ± 4	<.001
FEV_1_/FVC	73 ± 1	58 ± 2	<.001
BMI (kg/m^2^)	24.2 ± 0.8	25.3 ± 0.9	ns
CRP (mg/L)	15 {6–53}	6 {3–16}	<.05
WBC (G/L)	12.4 ± 0.7	11.2 ± 0.6	ns
neu (G/L)	10.1 ± 0.7	7.8 ± 0.5	<.05
ly (G/L)	1.6 ± 0.1	2.2 ± 0.1	<.05
neu/ly ratio	6.7 ± 0.7	4.5 ± 0.4	<.01
monocytes (G/L)	0.6 ± 0.0	0.7 ± 0.1	ns
hemoglobin (g/L)	131 ± 2	141 ± 3	ns
platelets (G/L)	367 ± 29	330 ± 16	ns
median PFS (months)	3.3	6.1	<.05
median OS (months)	11.0	15.0	ns

BMI: body mass index, WBC: white blood cell count, neu: neutrophil, ly: lymphocyte, CRP: C-reactive protein concentration, ^1^no statistical difference among various groups

## Discussion

Long-term smoking can induce chronic inflammation of lung parenchyma and the airways. Among many other cells Th17 [35, 36] and CD8^+^ [37] lymphocytes, as well as neutrophils [38] play major roles in development of smoking-induced chronic inflammation. In many individuals who are genetically unprotected from smoke-induced lung damage chronic obstructive pulmonary disease (COPD) develops [16]. Hogg et al. [39] have established important roles of CD4 and CD8 T cells in the process connecting heavy smoking with development of COPD. Smoking also increases the risk of lung cancer [13], and many lung cancer patients at the time of diagnosis have already suffered for years from COPD [15]. It has been deeply investigated how various elements of the pathogenesis of COPD contributed to pathogenesis of lung cancer. Recently, the shift from Th1 to Th2 polarization of the immune response [40], the accumulation of IL-17 producing Th17 lymphocytes [41], the simultaneous overproduction of certain cytokines, chemokines and matrix metalloproteinases [42] have been raised as possible elements of carcinogenesis in those who smoke and have suffered from COPD previously.

Of note, symptoms and laboratory signs of systemic inflammation characterize advanced NSCLC [20, 21]. Considering that stable COPD is a chronic inflammatory condition [25, 26, 37], concomitant COPD could synergize with tumor induced acute inflammation and worsen survival. This has been previously studied in early stage (I-IIIA) NSCLC patients having had undergone surgical resection of the tumor [43, 44]. In these studies, OS was found shorter in those who suffered also from COPD. However mechanisms of interference between COPD and NSCLC may be very different in curatively and only palliatively treatable NSCLC. While after curative treatment of early-stage NSCLC the expected OS streches to several years, after palliative treatment of advanced NSCLC median OS is only about 12 months [2]. Furthermore, in addition to many negative effects of concomitant COPD in cancer [43, 44], COPD is also keeping multiple antiinflammatory and antioxidant mechanisms active [45–47] prior to and probably during tumor progression. α1-antitrypsin, α2-macroglobulin, haptoglobin, orosomucoid [45], as well as ferroxidase (coeruloplasmin), surfactant protein–D [46] and glutathione peroxidase [47] become increased in sputum or plasma of COPD patients. These humoral antiinflammatory factors are supported by more abundant CD4 and CD8 T-lymphocytes in airways of COPD patients [37]. Moreover, in bronchial walls of such patients tertiary lymphoid tissues containing B-cells and immunoglobulin-producing plasma cells are characteristic [48]. These antiinflammatory and lymphoid mechanisms could, at least in theory, moderate tumor-induced inflammation and improve the effect of therapy in advanced NSCLC+COPD patients. Indeed, Mark et al. [14] have described a novel Th1 signature in resected pulmonary and tumor tissue of patients with COPD+NSCLC. The pulmonary Th1 response of COPD permeated the tumor microenvironment in these patients. These data supported the general notion that heavy smoking potentiates the efficacy of immune checkpoint inhibitor anticancer drugs in NSCLC [49]. Moreover, Mark et al. [14] could confirm that relative to non-COPD patients, tumor tissue of COPD patients contained more PD-1 and TIM3 (immune checkpoint) positive, exhausted CD4 cells. Further, advanced NSCLC+COPD patients responded with longer PFS versus non-COPD counterparts on immune checkpoint inhibitor treatment [14].

Some earlier studies investigated how concomitant COPD influenced the efficacy of chemotherapy in advanced NSCLC. In general, concomitant COPD exerted no deleterious influence on survival in any of these series. In a relatively small retrospective study concomitant COPD was found not to influence patient ratios with partial response, stable or progressive disease along first-line combined chemotherapy [10]. In another study [11] the overall response rate (ORR) was numerically higher among concomitant COPD that non-COPD NSCLC patients (38.9 vs 22.9%, *p* = 0.14), but OS remained unchanged. Of note, NSCLC+COPD patients were significantly elder (by 4 years (*p* < 0.001) than patients without COPD. Izquierdo et al. [12] have also demonstrated similar OS in concomitant COPD and non-COPD NSCLC patients, but in this study again mean age of the former group was 70 vs 66 years in the latter (p < 0.001). Arca et al. [13] have confirmed a prolongation of OS among COPD+NSCLC (*n* = 396) vs only NSCLC patients (*n* = 600) though the former group was older again by 3.5 years. These authors concluded that COPD increases OS of advanced lung cancer patients, but did not raise any hypothesis for explanation.

We have shown here in 175 advanced NSCLC patients that concomitant COPD did significantly prolong PFS after first-line chemotherapy. Some possible background mechanisms were revealed. NSCLC+COPD patients presented with a lower neutrophil/lymphocyte ratio, which was known as a sign of moderated tumor-induced inflammation and/or less inhibited antitumor immune response [7, 8]. Indeed, lower pretreatment neutrophil/lymphocyte ratio was shown by Yao et al. [7] to increase PFS in advanced NSCLC after first-line chemotherapy. Teramukai et al. [4] made similar observations by demonstrating longer PFS and OS in advanced NSCLC when pretreatment neutrophil count was lower than median. Lee et al. [50] documented that early reduction of the neutrophil/lymphocyte ratio was a surrogate marker of better OS in advanced adenocarcinoma of the lung treated by gefitinib or standard chemotherapy inthe first-line. The ratio of patients suffering also from COPD was not determined in these works.

Earlier studies have demonstrated [23, 24] that the analysis of exhausted and reinvigorated T cells could be performed in peripheral blood of cancer patients. Our present finding of increased granzyme B + CD8 fraction in NSCLC+COPD patients relative to only NSCLC has also pointed to less inhibited immune responsiveness in advanced NSCLC if associated by COPD. We also found here that concomitant COPD interfered with various known myeloid and lymphoid effects of VEGF. Higher VEGF concentrations were found to be statistically significantly associated with more M-MDSC-s and neutrophil granulocytes in NSCLC, but not in those patients who had concomitant COPD. Moreover, only among the latter was direct relationship revealed between VEGF and lymphocyte count. VEGF was demonstrated to stimulate VEGFR2-induced signals within T cells [49]. It is possible therefore, that such mechanisms could play a role in making NSCLC more responsive to treatment in those patients who also had COPD.

This study had limitations. First, airway obstruction was more severe in only COPD than in NSCLC+COPD patients. However, this way the immunologic and inflammatory impact of concomitant COPD in NSCLC+COPD patients has probably not been overinterpreted. Cytokines and various immune cell populations were not measured in all but subsequents subgroups of NSCLC and NSCLC+COPD patients. However, more important data like PFS, neutrophil and lymphocyte counts, the neutrophil/lymphocyte ratio and CRP concentration have been supported by whole group data.

## Conclusions

This work indicates that COPD occurring concomitantly with advanced NSCLC improves the efficacy of first line chemotherapy. This beneficial effect of COPD may be mediated by reduced tumor-induced inflammation and a less impeded antitumor immune response. Heavy smoking has been shown to increase the number of tumor-associated mutations, thereby the efficacy of immune checkpoint inhibitors in NSCLC [51, 52]. But chemotherapy has also been known to induce tumor cell expression of MHC-I [53] and reduce regulatory T cells and MDSC-s [54]. Therefore, concomitant COPD may support immunologic anticancer mechanisms induced by both chemotherapy and immune checkpont inhibition.
